# Severe hypokalemia, metabolic alkalosis and hypertension in a 54 year old male with ectopic ACTH syndrome: a case report

**DOI:** 10.4076/1757-1626-2-6174

**Published:** 2009-07-31

**Authors:** Miguel Angel Martínez-Valles, Asael Palafox-Cazarez, Jose Antonio Paredes-Avina

**Affiliations:** Departamento de Medicina Interna, Hospital RegionalDr. “Valentín Gómez Farías” Instituto de Seguridad y Servicios Sociales de los Trabajadores del Estado. Av. Soledad Orozco 203, Col. EL Capullo, Zapopan, 45150, JaliscoMexico

## Abstract

Ectopic ACTH syndrome is a rare cause of Cushing’s syndrome accounting for about 15% of all cases. Small cell lung cancer and bronchial carcinoids account for about half of the cases. Malignant neoplasm has rapid and more aggressive metabolic effects. We report a 54-year-old male patient with phenotypic features of Cushing’s syndrome with severe hypokalemia, metabolic alkalosis, hypertension and altered mental status as manifestations of an ACTH-secreting small cell carcinoma from the lung. Ectopic ACTH syndrome should be highly considered in patients with hypertension and severe hypokalemic metabolic alkalosis, especially when a lung mass is discovered.

## Introduction

Certain tumors of non-endocrine tissues can give rise to Cushing’s syndrome by secreting an adrenocorticotropic substance, a concept that was proposed by Meador et al. more than 50 years ago [1]. Ectopic ACTH secretion (EAS) is a very rare cause of Cushing’s syndrome accounting for about 15% of cases, and about 20% of ACTH-dependent cases. Virtually all tumors have been associated with EAS, however lung cancer (including small cell carcinoma and bronchial carcinoids) accounts for half of the cases and more than a quarter of the cases remains occult without determining the source of the ectopic secretion [2]. Differentiation between the two forms of ACTH-dependent Cushing’s syndrome is a challenging task that requires a well structured diagnostic work-up [3]. Duration of the disease and glucocorticoid hormone levels are major determinants in the clinical appearance of Cushing’s syndrome. The type of tumor strongly influences the clinical course: EAS by small cell carcinoma (SCC) and other malignant neoplasms typically leads to early appearance and more pronounced metabolic alterations. In contrast, less aggressive tumors with a delayed diagnosis show clinical characteristics similar to those of pituitary Cushing’s syndrome [4]. Clinicians should be aware that in a patient with rapidly progressive small cell lung cancer (SCLC) and a deteriorating clinical condition; the development of ectopic Cushing’s syndrome can pass undiagnosed, leading to significant additional comorbidity [5]. Hypertension with hypokalemia is a common finding in many endocrinological disorders, including Cushing’s syndrome. When a patient has a rapid deterioration in clinical condition with severe metabolic alkalosis and neuropsychiatric symptoms, EAS must be considered [5,6]. We report a case of a Mexican mestizo man with severe hypertension, hypokalemia, altered mental status and severe metabolic alkalosis as presentations of EAS by a small cell carcinoma from the lung.

## Case presentation

A 54 year old Mexican mestizo man was admitted to the Internal Medicine department of the Regional Hospital Dr. “Valentín Gómez Farías” presenting with altered mental status, paresthesias, hypokalemia, and severe hypertension. The patient worked as a teacher. He was a 10 pack year smoker for 45 years and an occasional drinker. He also has a history of using cocaine and smoking marijuana. He lived a sedentary life, and was obese since childhood, being unable to lose weight with conventional diets. He had no family history of cancer or surgical interventions.

**Figure 1.**
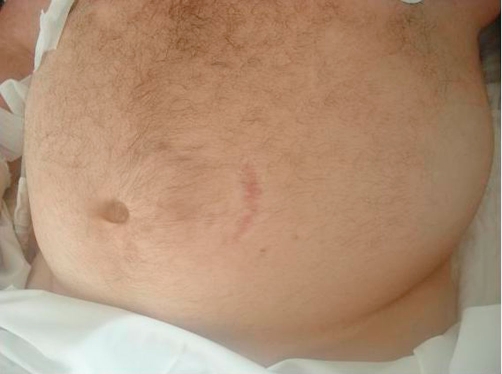
Abdominal stretch marks.

**Figure 2.**
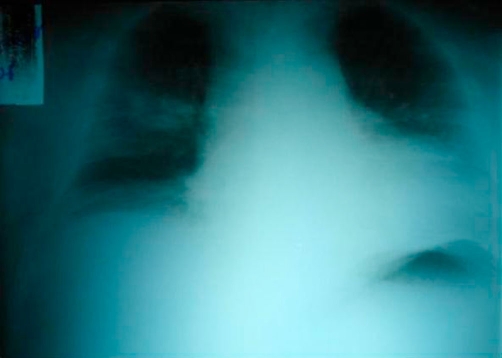
Chest X-ray showing a diffuse nodular opacity in the hilar region of the right lung.

**Figure 3.**
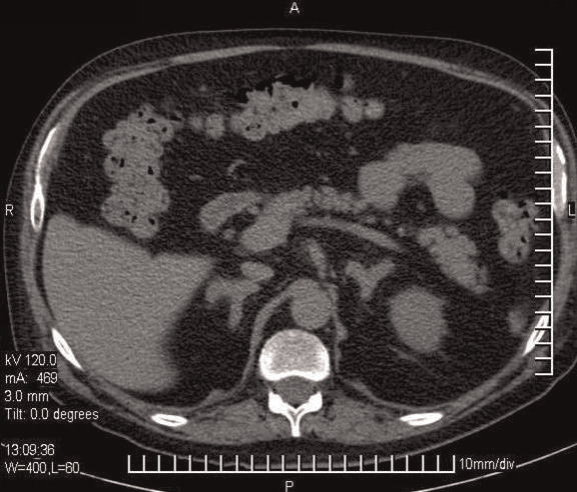
Abdominal CT scan showing diffuse enlargement (hyperplasia) of the adrenal glands without focal lesions. No metastatic lesions were observed in the liver and spleen. Also there was not lymph node enlargement.

**Figure 4.**
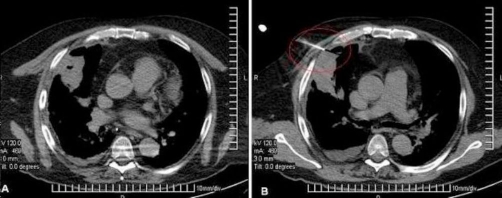
**(A)** Chest CT scan showing a cavitated right lung mass and right pleural effusion, **(B)** CT guided fine-needle lung biopsy.

**Figure 5.**
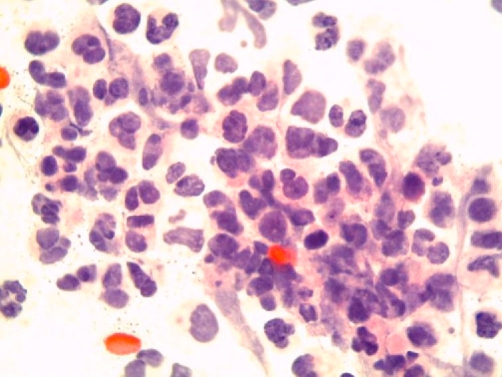
Photomicrography of the histology of the lung mass biopsy specimen (100×) showing atypical small cells with sparse acidophil cytoplasm, lobulated and rounded nuclei, and compact chromatin.

The patient was in good health until three months before admission when he presented with fatigue, intermittent paresthesias of all limbs, cold intolerance, polyuria, weight gain, and irritability. He consulted a primary care physician that made a diagnosis of diabetes mellitus, hypertension, and dyslipidemia and began treatment with Metformin 850 mg twice daily, Glargine insulin 16 units in the morning, Enalapril 10 mg twice daily, and Atorvastatin 20 mg at night. After one month, the patient discontinued the Enalapril on his own. During the next few months the patient presented with generalized edema, abdominal striae, and depressive symptoms. Also two days before admission he presented with confusion. On arrival, the physical exam found him to be obese, with plethoric facies, severe edema of the lower limbs, bruising and red-purple abdominal striae (Figure 1). He was confused without any sign of focalization or lateralization. His vital signs showed a blood pressure of 210/140 mmHg, heart rate of 62 beats per minute, respiratory rate of 14 per minute, and temperature of 37.2 degree Celsius. Blood biochemistry results were as follows: hypokalemia of 1.8 mEq/L with severe metabolic alkalosis (ph 7.59, HCO3 50.7, pO2 50, Sat O2 90%), white cell count 5740/mmc, hemoglobin 13.1 g/dL, platelets 161,000/mmc, glucose 198 mg/dL, creatinine 0.74 mg/dL, sodium 141 mEq/L, chloride 99 mEq/L, lactic dehydrogenase 702 U/L, ALT 26.7 U/L, CPK 91 U/L, albumin 3.2 g/dL, PT 12 seconds (test 12.4 seconds) INR 0.95, urinary sodium 45.9 mmol/L, urinary potassium 48.2 mmol/L, urine glucose +++, urinary proteins +. The electrocardiogram showed a first degree AV block. The lungs where clear under auscultation. The chest X-ray showed an undefined nodular opacity in the hilar region of the right lung (Figure 2). We started intravenous administration of large amount of potassium chloride (20 mEq per hour), intravenous insulin, and an intravenous antihypertensive (sodium nitroprusside). Following the treatment he experienced complete remission of mental confusion, and improvement, however his blood pressure did not normalize (150/100 mmHg). In view of these findings a protocol for detecting endocrine hypertension was followed, including tests for Cushing’s syndrome and primary hyperaldosteronism. Forty-eight hours later basal plasma renin activity and aldosterone were measured and determined as normal. The respective values were 0.53 ng/ml/h (normal values between 0.2-2.8 ng/ml/h) and 52.5 pg/mL (normal values between 10-160 pg/mL). The abdominal CT scan showed diffuse enlargement of the adrenal glands without focal lesions, without any abnormalities of the liver or spleen (Figure 3). The 24 hour free cortisol urinary levels were 6600 μg (normal values 4-100 μg) using the immunoenzymatic method. The ACTH levels were 107 pg/mL (normal values are less than 46 pg/mL), by the quimioluminiscence method. High dose dexamethasone suppression test (8 mg) showed suppression of approximately 8% of the cortisol serum levels. A brain CT scan, including the sellar region was normal. These results, together with the clinical and radiological findings support the diagnostic hypothesis of ectopic ACTH-dependent Cushing’s syndrome. The patient persisted with hypokalemia and hypertension, both of which could not be controlled despite management with large dose of intravenous potassium (up to 240 mEq per day) for the hypokalemia, and Enalapril 20 mgs twice daily, Telmisartan 160 mg four times daily, Felodipine 10 mgs twice daily, and Spironolactone 200 mg four times daily (once primary hyperaldosteronism was excluded) for the hypertension. The concentration of tumor markers were as follows: alpha-fetoprotein of 3.8 ng/ml (normal values 0-15 ng/ml), carcinoembryonic antigen of 2.6 ng/ml (normal values 0-5 ng/ml), and serum glycoprotein CA 125 of 10.96 U/ml (normal values 0-35 U/ml). A chest CT scan showed a cavitated right lung mass localized in the medial lobe of the anterior medial segment and minimal pleural effusion (Figure 4A). We also observed some degree of pleural thickening. On day 24 a CT guided fine-needle lung biopsy was performed, (Figure 4B) showing histopathological results of a small cell neoplasm (Figure 5). The instability of the patient did not allow for the initiation of chemotherapy, therefore treatment with Ketoconazole 400 mgs was begun before initiating chemotherapy. While waiting for the patient’s basal situation to improve before starting chemotherapy, we started ketoconazole 400 mgs twice daily with a rapid normalization of blood pressure and potassium plasma levels. Seventy two hours later the patient presented with sepsis due to a right leg cellulitis that was treated with meropenem. Despite a partial response to the medical treatment the patient worsened and developed bilateral pleural effusions, and respiratory failure that required assisted mechanical ventilation dying a few days later.

## Discussion

The case reported here was presented as a hypertensive crisis associated with hypokalemia, severe metabolic alkalosis and neuropsychiatric symptoms. The patient’s initial symptoms are explained by the presence of metabolic abnormalities due to severe hypercortisolism (diabetes mellitus, hypertension and hypokalemia) but the initial diagnosis was delayed due to poor clinical suspicion and a high prevalence of diseases such as diabetes and hypertension in Mexico. The rapid deterioration of his health, and some cushingoid characteristics, strongly suggested that the clinical symptoms were secondary to some cause of Cushing’s syndrome [5].

Cortisol increases the blood pressure by multiple mechanisms. The high blood pressure upon arrival (due to his poor adherence to treatment and hypercortisolism) and the metabolic abnormalities explain the neurological symptoms like confusion seen in this case. Once the blood pressure of the patient decreased, and large amounts of potassium were administrated, a rapid improvement in his neurological status was observed. However, the patient continued to demonstrate minor depressive symptoms (mainly apathy, hypersomnia, irritability, and hopeless thoughts). In patients with EAS, psychiatric symptoms are frequently reported in about 50% of cases. The most frequent are depression and psychosis [6]. Although the association of hypertension and hypokalemia are often attributed to primary hyperaldosteronism, the normal levels of plasma renin activity and aldosterone are opposed in this diagnosis. But in view of the mineralocorticoid action of high cortisol concentrations, other causes should be considered. The local cortisol conversion to cortisone by the action of 11 beta-hydroxysteriod dehydrogenase is the rate-limiting step for the mineralocorticoid effect of cortisol under normal conditions. When plasma concentrations of cortisol are very high, the action of this enzyme is insufficient (the mineralocorticoid escape phenomenon) and mineralocorticoid effects appear [2]. The elevated levels of ACTH, free urinary cortisol excretion, a positive high dexamethasone suppression test and the hilar opacity observed in chest X-ray strongly suggested that the clinical symptoms were due to a malignant ACTH secreting neoplasm.

The gold standard for the differential diagnosis of pituitary and ectopic ACTH-dependent Cushing’s syndrome is the bilateral inferior petrosal sinus sampling after administration of CRH (1 μg/kg). A ratio greater than 3.0 after the administration of CRH is consistent with Cushing’s disease. Patients with EAS will have a ratio less than 2 before and after CRH administration because the endogenous hypercortisolism suppresses pituitary ACTH release through negative feedback mechanisms (sensitivity 97%, specificity 100%). High-dose dexamethasone suppression tests have lower sensitivity (81%) and specificity (67%) [3]. In this case, the rapid clinical course did not permit pituitary magnetic resonance imaging or the tests generally considered as gold standards for ectopic ACTH-dependent Cushing’s syndrome (although the CT scan from the sellar region did not reveal visible lesions). Nevertheless, we can establish this diagnosis with high probability, since this patient’s Cushing’s syndrome was ACTH-dependent, with a positive result in the high-dose dexamethasone suppression test with characteristic phenotype and biochemical presentation (hypertension and severe hypokalemic metabolic alkalosis), and with a tumor type that can cause Cushing’s syndrome.

The SCLC is a well recognized origin of EAS. The incidence of SCLC is about 13% of all newly diagnosed lung cancers in Mexico [7], and patients with extensive disease have median survival duration of 6 weeks. The course seen in the present case is in line with this view. Although there are few cases of EAS reported in Mexico, the longest series of reported cases (8 patients) the lung was traced as the source with bronchial carcinoids being diagnosed in half [8]. Because the patient’s rapid deterioration no more diagnostic studies were performed. An autopsy was not performed.

Ketoconazole has been used to treat Cushing’s syndrome by inhibiting adrenal glucocorticoid synthesis. Treatment with ketoconazole promotes a palliative hormonal response in more than 50% of patients [9]. In this patient, ketoconazole (400 mg twice daily) promoted a rapid resolution of hypokalemia and hypertension. Due to a rapid clinical deterioration, the patient was not able to receive standard chemotherapy with carboplatin and etoposide for SCLC [10].

## Conclusion

Ectopic ACTH syndrome is a rare cause of ACTH-dependent Cushing syndrome. Nearly any neuroendocrine or non endocrine tumor may be associated with EAS. In advanced tumors the features of hypercortisolism may be masked by a general deterioration in clinical condition and interpreted as a progression of the neoplastic disease. Failure to recognize EAS can carry additional morbidity. EAS requires a well established diagnostic work up, and must be considered strongly in patients with hypertension and severe hypokalemic metabolic alkalosis especially when a lung mass is discovered.

## Abbreviations

ACTH: Adrenocorticotropic Hormone
CRH: Corticotropin-releasing hormone
CT: Computed Tomography
EAS: Ectopic ACTH Syndrome
SCLC: Small Cell Lung Cancer

## References

[bib-001] Meador CK, Liddle GW, Island DP, Nicholson WE, Lucas CP, Nuckton JG, Luetscher JA (1962). Cause of Cushing’s syndrome in patients with tumors arising from nonendocrine tissue. J Clin Endocrinol Metab.

[bib-002] Wajchenberg BL, Mendonca BB, Liberman B, Albergaria Pereira MA, Campos Carneiro P, Wakamatsu A, Kirschner MA (1994). Ectopic adrenocorticotropic hormone syndrome. Endocr Rev.

[bib-003] Hershel Raff, Findling James W (2003). A physiologic approach to diagnosis of the Cushing syndrome. Ann Intern Med.

[bib-004] Isidori Andrea M, Andrea Lenzi (2007). Ectopic ACTH syndrome. Arq Bras Endocrinol Metab.

[bib-005] Isidori Andrea M, Kaltsas Gregory A, Carlotta Pozza, Vanni Frajese, John Newell-Price, Reznek Rodney H, Jenkins Paul J, Monson John P, Grossman Ashley B, Michael Besser G (2006). The ectopic adrenocorticotropin syndrome: clinical features, diagnosis, management, and long-term follow-up. J Clin Endocrinol Metab.

[bib-006] Ioannis Ilias, Torpy David J, Karel Pacak, Nancy Mullen, Wesley Robert A, Nieman Lynnette K (2005). Cushing’s syndrome due to ectopic corticotropin secretion twenty years’ experience at the national institutes of health. J Clin Endocrinol Metab.

[bib-007] Frumencio Medina M, Margarita Salazar F, Cecilia García S, Francisco Franco M (2002). Epidemiología descriptiva del cáncer pulmonar en el Instituto Nacionale de Enfermedades Respiratorias México 1997-2000. Rev Inst Nal Enf Resp Mex.

[bib-008] Irma Hernandez, Ana Laura Espinoza-de-los-Monteros, Victoria Mendoza, Sonia Cheng, Mario Molina, Ernesto Sosa, Mercado Moises (2006). Ectopic ACTH-secreting syndrome: a single center experience report. Arch Med Res.

[bib-009] Winquist EW, Laskey J, Crupo M, Khamsi F, Shepard FA (1995). Ketoconazole in the management of paraneoplastic Cushings syndrome secondary to ectopic adrenocorticotropin production. J Clin Oncol.

[bib-0010] Kosmidis PA, Samantas E, Fountzilas G, Plavdis N, Apostolopoulou F, Skarlos D (1994). Cisplatin/etoposide versus carboplatin/etoposide chemotherapy and irradiation in small cell lung cancer: a randomized phase III study. Semin Oncol.

